# How many general practice consultations occur in Ireland annually? Cross-sectional data from a survey of general practices

**DOI:** 10.1186/s12875-021-01377-0

**Published:** 2021-02-20

**Authors:** C. Collins, R. Homeniuk

**Affiliations:** Irish College of General Practitioners, Dublin, Ireland

**Keywords:** General practice, Consultation rates, General practitioner, Practice nurse

## Abstract

**Background:**

General practice plays a central role in the Irish health system. This study aimed to determine a baseline estimate of the number of consultations completed in general practice in Ireland annually to facilitate evidence-based healthcare planning.

**Methods:**

A survey was emailed to all 3378 individual members of the Irish College of General Practitioners in February 2020 asking each practice to report on consultations by general practitioners and practice nurses occurring on the previous day of practice.

**Results:**

A total of 507 practices responded to the survey, reporting 34,594 general practitioner consultations and 13,161 nurse consultations on one day. Extrapolating this figure nationally, we estimate 21,353,731 GP consultations and 7,749,615 practice nurse consultations occur in Ireland annually. An Irish person visits their GP an estimated 4.34 times a year and the average consultation time is 13.7 min.

**Conclusions:**

This study shows that over 29.1 million consultations take place in Irish general practice every year. Innovative interventions to increase the capacity of general practice are needed to ensure high-quality care continues to be accessible in Ireland.

**Supplementary Information:**

The online version contains supplementary material available at 10.1186/s12875-021-01377-0.

## Background

Healthcare is a constantly changing landscape, with many factors leading to increased demand for general practice services, such as population growth and ageing, policy measures, and the increased prevalence of multi-morbidities [1]. General practice plays a central role in every health system [2].

Approximately 43% of the Irish population qualify for free access to general practice care as holders of either a General Medical Services (GMS) card (32.4%) or general practitioner (GP) only card (known as a doctor visit card -DVC) (10.4%), with the remainder paying privately per visit to their GP [3]. The GMS scheme entitles patients with incomes below a threshold on a means tested basis or with specified illnesses to free health care including medication, while a DVC provides free GP access only. The transition from a mixed public and privately funded health system to a system of universal healthcare began in Ireland in 2015 when those aged under six and over 70 years became entitled to DVCs [4].

With the introduction of free care to people under six and over 70 years and plans to roll out free GP services to all citizens [2], understanding practice activity and workforce issues within general practice is essential for healthcare planning.

However, due to a lack of a central register for GPs and the nature of practice in Ireland with GPs operating independently, information about both GP numbers and the volume of general practice consultations is difficult to ascertain [2, 5]. GPs operate as private professionals and while almost all are computerised, there is no national data reporting system or data extraction for general practice data. The professional body for GPs in Ireland, the Irish College of General Practitioners (ICGP), estimates that 3496 GPs are actively practising in Ireland [6], both in general practices and out-of-hours facilities, with 25% of these working on a part-time basis. This number is similar to other estimates, such as 4250 GPs from the OECD and 4784 registered with the Medical Council [6]. The smaller estimate from the ICGP excludes GPs who are retired but still registered, those who reported working abroad and those still in training. It is estimated that around 25% of GPs in Ireland are currently working in single-handed practices [5].

Since 2015, past estimates of the average number of consultations were determined with the Annual Healthy Ireland survey [7] and suggest that people aged 15 and older visited a doctor (including consultants) on average 5.8 times a year in 2019 [8]. European statistics reported by the OECD [9] and Eurostat [10] state that in 2018, the average number of GP consultations per inhabitant in Ireland was 5.04, a reduction from 5.75 in 2016. However, it should be noted that in the OECD doctor consultation dataset, which spans decades, Ireland only had information included for five years, highlighting the lack of documentation of health care utilisation in Ireland historically. In our study, we measured consultations conducted by general practitioners and practice nurses.

The need for consistent and up to date data about GP practice activity in Ireland is evident from previous publications [2, 5, 7, 11]. One of the recommendations from the national health and social care coordination centre - the Health Service Executive (HSE) - medical workforce planning report was ‘A national register of GPs should be introduced to improve the availability and quality of data on the GP workforce’ [2]. This recommendation has become a critical requirement in light of Ireland’s policy to move more healthcare into the community [12]. The Department of Health released a healthcare capacity review in 2018 [13] which estimated that in order to meet rising demand and reform health services, the primary care workforce needs a 48% increase by 2031 – with required increases of 39 and 89% for GPs and PNs respectively [13]. Workforce and consultation data are necessary in order to determine capacity and requirements for our future healthcare system.

In February 2020, the ICGP sent an online questionnaire on practice activity to its membership to help fill this information gap. This paper aims to present data from this survey regarding the number and nature of consultations taking place. The results we present may act as a baseline for GP activity and enable evidence-based decisions for healthcare planning in Ireland.

## Methods

At the beginning of February 2020, a survey to estimate consultation rates in general practice was sent to 3378 ICGP members. This excludes GPs registered in Ireland who are working abroad, trainees, retired GPs who are still registered, and those who are not ICGP members. Only one response per practice was requested. There are an estimated 1635 practices in Ireland with 75% of these being group practices [5]. Practices were requested to provide details on the number of full-time equivalent (FTE) doctors and practices nurses (PNs) working in the practice and the number and type of consultations completed by both GPs and PNs on the relevant working day. The questionnaire was developed specifically for this survey and is included as an additional file.

To estimate national consultation rates for Ireland, we extrapolated our data by using mean consultations completed by GPs daily and multiplying that by working days (Monday to Friday) in a year and number of GPs working, based on available estimates [6]. Our estimate includes face-to-face consultations, telemedicine consultations, home visits and visits to nursing homes reported by practices who responded to the survey. If any consultation type was unanswered i.e. missing data, this was included in the total consultation calculation for the practice as zero. Data was returned about the most recent working day. The total number of consultations reported for GPs and PNs and the number of GPs and PNs working on the day was used to calculate mean consultations per GP/PN daily, and the overall figure was used in the annual estimate calculation as explained above. Out of hours services in Ireland are provided and recorded separately, hence consultations after 6pm Monday to Friday and at weekends are not included in these figures. General practices in Ireland operate as private businesses and hence GPs generally employ locums or have a colleague cover when they take leave – 83% of GPs being present on any working day is based on the proportion of GPs employed in the practice who were working on the day data were reported on. The Department of Health has previously reported just under one million out-of-hours GP contacts in 2019 [3]. To calculate the average number of visits to general practice per person, we used the population figure reported by the Central Statistics Office in 2019 – which is 4,921,500 [14]. We divided the total number of consultations by GPs by the population to estimate the number of visits per person per year. To estimate average length of appointment, we removed three hours from the average working hours reported by GPs to account for the average time spent on admin daily [6]. The remaining time was divided by mean consultation numbers and converted into minutes. We conducted analysis using SPSS V25 software, using descriptive analysis. For numerical data, means were used for comparisons and to conduct statistical tests as appropriate. Average consultation numbers are based on the number of consultations provided by the survey, and workforce information from previous research [5, 6, 13] was used to extrapolate findings. Outliers for consultations and hours worked data were defined as those outside 1.5 times the interquartile range and were removed from calculations. Chi-square tests were used for categorical comparisons. Pearson’s and Spearman’s tests were used to test for correlation between continuous variables. T-tests and F-tests were used to compare means as appropriate.

## Results

Overall, 526 practices completed the questionnaire representing 32% of all practices in Ireland. After removing outliers, 507 responses were included. In total, 1456.6 full time equivalent (FTE) GPs worked in the responding practices with an average of 2.9 FTE GPs in each practice. Overall, 18% of practices were single-handed. On the day, there were between one FTE and eight FTE GPs on duty. In our sample, 43.6% of practices responding were located in towns, 37.5% in cities and 18.9% in villages. At least one practice was recorded for every county in Ireland; one third of practices who responded were located in Dublin, and the next biggest concentrations were Cork (13%) and Galway (8%) which is similar to the population distribution.

Out of our responding practices, 94% (*n* = 497) employed a practice nurse on at least a part-time basis. Just under two-thirds (62%) of these practices had at least one FTE PN. Overall, 603.5 FTE PNs were employed across 497 responding practices. There was a range of zero to eight total PNs employed at practices in our sample, with the average being 1.2 FTE PNs per practice overall.

After outliers were removed, GPs reported working between six and 13.5 h (including non-clinical work), with an average of 9.7 daily work hours. Out of the total FTE GP workforce represented in the survey, 83% (1214.3) FTE GPs were working on the day when consultations were recorded. For nurses, 85% of the reported workforce was present on the day data was recorded, with 510.3 FTE PNs working on the day. These figures were based on the closest clinical day to survey completion.

**Fig. 1:**
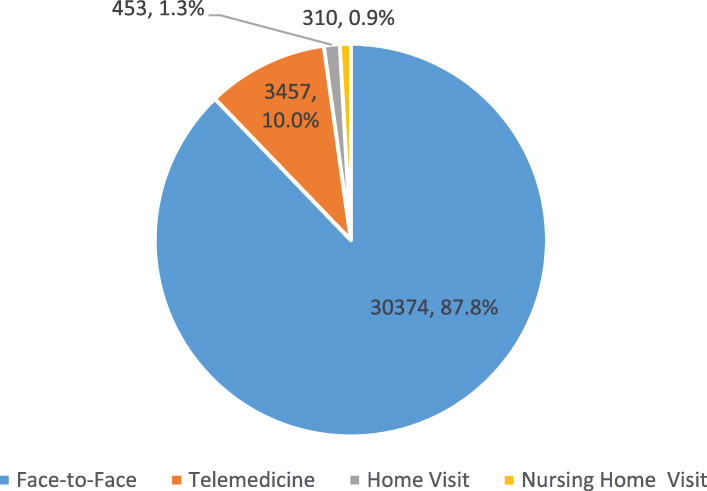
Breakdown of GP Consultation types

**Fig. 2:**
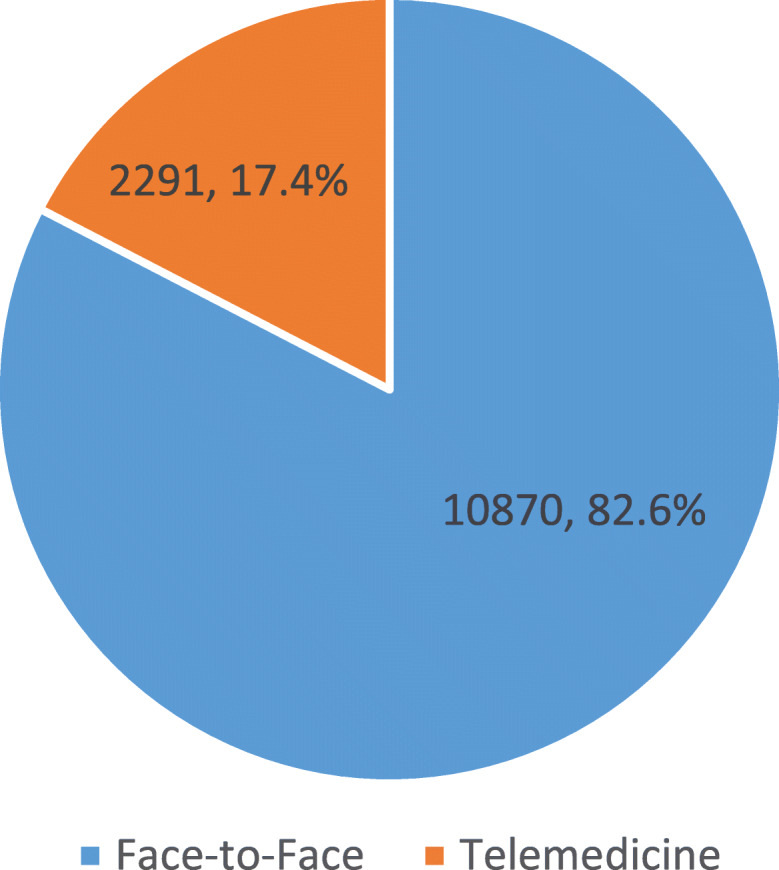
Breakdown of PN Consultation types

Overall, 507 practices reported 34,594 GP consultations on the day in respect of the 1214.3 FTE GPs working. Some practices neglected to answer some questions; responses ranged from 457 to 507 practices. This consultation figure includes face-to-face (*n* = 507) and telemedicine appointments (*n* = 490) along with visits to homes (*n* = 492) and nursing homes (*n* = 479). The majority of consultations provided were face-to-face (87.8%), with telemedicine, home visits and nursing home visits making up 10.0, 1.3 and 0.9% of appointments respectively. Figure 1 shows the full breakdown of consultations completed by GPs on a day in February 2020 and Fig. 2 shows the breakdown of consultations completed on the day by PNs in the sample.

**Fig. 3:**
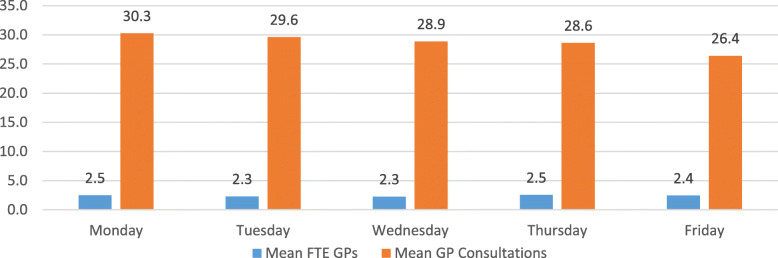
Mean Full-time equivalent GPs and Mean Consultations per FTE GP on the days when data was recorded

Responses were received for each day of the week; however, 39.4% of responses reported data for Monday, with a gradual decline towards the end of the week. The average number of FTE GPs working on the day per practice was 2.4, which varied from 2.3 to 2.5 on different days of the week. The variation in mean consultations per GP per day was not found to be statistically significant, with a difference of 3.8 consultations between highest and lowest daily consultation rates. In Fig. 3, the average number of FTE GPs on the day and the mean consultations completed per GP each day are shown.

Sixty-two percent of responding practices (*n* = 490) had telemedicine in place, with both nurses and GPs making use of this technology. Nearly one-fifth (17.4%) of appointments conducted by nurses were completed using telemedicine. Nurses contributed 27.6% of all consultations recorded in our survey, with 13,161 consultations completed by nurses. This figure includes both face-to-face and telemedicine consultations.

GPs at single-handed practices had an average consultation rate of 32 daily, 11.0% of these consultations occurring via telemedicine. Comparatively, GPs at group practices had an average of 29 consultations a day with 9.9% being telemedicine appointments. There was a statistically significant difference between the mean consultation rate per GP dependant on practice size (*p < 0.05).* Considering an average of three hours of admin duties [6]; GPs overall worked an average of 9.7 h and spent an average of 13.7 min per consultation. Using Pearson’s test for correlation we found statistically significant correlations between the number of GP consultations (all types) and having a PN working on the day and with having a PN employed at the practice even if not working on the day in question (*p* = 0.01). However, no significant relationship was found between employing a PN or having a PN working on the day and the hours worked by GPs.

We extrapolated our findings to create national estimates of the number of consultations conducted. Eighty-three percent of the overall GPs in our sample of 507 practices were working on the day consultation numbers were recorded. Hence, in our calculations, we assumed that on any given working day 83% of practising GPs in Ireland are working. In order to reflect usual working hours and capacity of general practice, this estimate used 253 working days.

According to our responses, a GP completes an average 29 consultations per day. Applying these statistics, based on 83% of 3496 [6] GPs working each of 253 working days and completing an average of 29 consultations, there are 84,402 consultations daily, totalling 21,353,731 per annum. When considering the number of visits across 4,921,500 people living in Ireland [14], we find an estimated 4.34 GP visits per year per person. This covers the whole population, including those under 15, who are not normally included in these calculations; however, due to not having patient demographics in our data, we cannot stratify for different age groups. This also does not take into account any seasonal differences in GP activity.

**Fig. 4:**
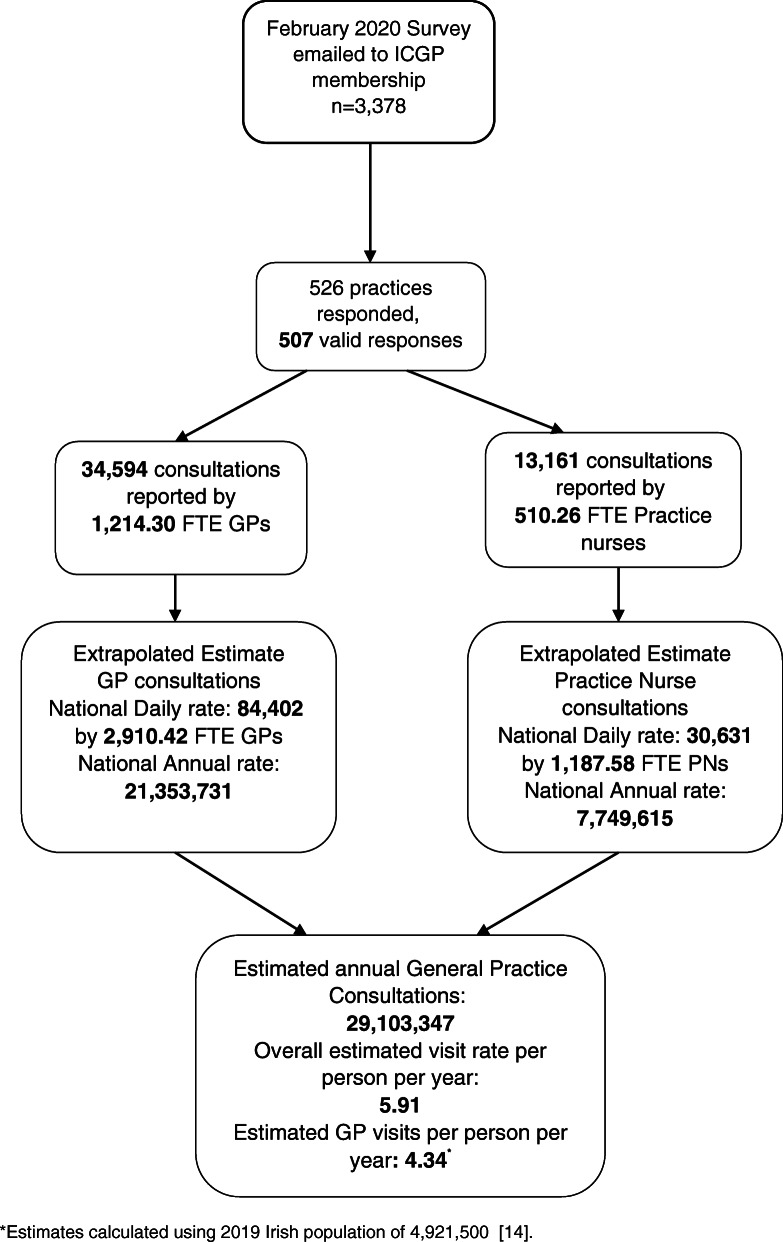
Consultations Flow Chart

The study included 601.5 FTE PNs in total, with 85% (510.3) available on the day when consultations were counted. In total, they completed 13,161 consultations; 17.4% of these occurred via telemedicine. The average number of consultations by PNs daily was 26 each. There are an estimated 1400 FTE practice nurses in Ireland [13]; by using the estimated 85% present on the day of recording, our national estimates are 30,631 PN consultations daily, which equals 7,749,615 annual consultations using the same 253 normal working days as above. Figure 4 shows the estimated flow of primary care capacity and consultation output.

In total, we estimate 29,103,347 consultations occur each year in general practice across GPs and PNs. We estimate an average Irish person visits general practice (GP and practice nurse consultations combined) 5.91 times per year.

## Discussion

### Summary of key results

In this study, we estimated the number of consultations that occur in general practice in Ireland. With a sample of 507 practices, 34,594 GP consultations and 13,161 PN consultations, we extrapolated these figures to estimate that 21.4 million GP consultations and 7.7 million PN consultations occur annually. By using the 2019 Irish population figure of 4.9 million [14], we estimate that people visit a GP 4.34 times a year. Using the same calculation for the population, the figure increases to 5.91 general practice visits per person per year when both GP and PN visits are included.

We found differences in the average number of consultations per GP carried out at single-handed practices compared to group practices. Single-handed GPs had an average consultation rate of 32 per day compared to 29 per day for GPs in group practices; this difference was found to be statistically significant (*p <* 0.05).

In our sample, only 5.8% of practices did not employ a PN on at least a part-time basis. PNs completed an average of 26 consultations a day and conducted over a quarter (27.6%) of all consultations. Overall, 17.4% of nurse consultations were conducted via telemedicine compared to only 10.0% for GPs.

### Comparison to the literature

Between 2015 and 2019, the proportion of single-handed GP practices in Ireland varies from 18 to 25% [5, 11], which is consistent with the 18% found in our responding sample.

In 2015 and 2019, around 82% of practices were found to employ a practice nurse on at least a part-time basis [5, 13], which is comparable to our findings.

Our estimate of annual GP consultation rates in Ireland sits between other estimates from 2013 to 2019. The Department of Health’s health capacity report estimated 18.9 million GP consultations in 2016 could rise to 26.2 million in 2031 and PN consultations could go from 6.8 million in 2016 to 9.5 million in 2031 [13]. The 2019 ESRI estimate was 17.5 million GP consultations [5] and Behan et al.’s 2013 estimate (which includes out of hours consultations) was 24.6 million [15]. While all three are similar, the lack of nationally available data about general practice activity means that it is hard to have an accurate count of consultations and data are not directly comparable. In the Department of Health report, a purposively designed demand and capacity model was employed making use of data from over seven sources including CSO and HSE data [13]. The ESRI estimates [5, 16] are based on amalgamating various sources regarding GP supply, patient usage and CSO statistics. Behan et al. [15] includes out of hours consultations and covers one full year of data collected retrospectively at one point in time from six practices.

The Organisation for Economic Co-operation and Development (OECD) reported an average of 5.0 annual consultations per person in 2018 [9] which equates to 24.1 million annual consultations. Eurostat reported the same amount [10]. Most recently, the 2019 Healthy Ireland Survey [17] found that the average person saw their GP 4.5 times in a year, which is very close to our estimate of 4.34 visits per annum based on the number of consultations reported by GPs. However, all but two of these estimates used different methods to determine the consultation rates.

GPs told us they work an average of 9.7 h daily, which is consistent with other Irish findings [6, 17, 18]. This includes admin time, shown elsewhere to be an average of three hours per day [6, 18]. We estimated GPs spend an average of 13.7 min on a consultation, which is slightly lower but in line with findings of 14–15 min by others [18, 19].

### Contribution/implications for policy and research

It is important to understand health care utilisation for future health planning; accurate and routine data collection and trend analysis is essential to this understanding. From the data presented in this paper, it is clear that practice nurses have an important role to play in Irish general practice.

Usage of telemedicine is greater among practice nurses and therefore its use in Irish general practice pre-COVID-19 suggests it may be linked to the reason for consultation. According to the Irish Practice Nurse Association, PNs carry out a wide range of services which include nutrition management, smoking cessation, preventative care and self-management support [20]. Telemedicine delivery of many of these services has been found to have equivalent outcomes to face-to-face appointments [21]. As these services are included under the practice nurse’s scope in Ireland, this could be why they had a higher proportion of consultations using telemedicine.

Using the most recent European figures [9, 10], there was an increase in visits to doctors in 2015–2016, which could be related to the introduction of free GP services for those aged under six and over 70 years. However, this drops again by 2018 despite a growing population. All of these estimates are much higher than the 3.2 consultations per person reported by CSO in 2010 [15], again highlighting the need for a national data collection strategy to capture all consultations in general practice. Without this, accurate workforce predictions and planning will be very difficult.

Part of Irish national health policy [12] is an emphasis on better collection and use of data to inform decision-making and employ population-focused health planning. As shown by our results, standard, centralised methods and collection schedules need to be implemented for Irish general practice to accurately gauge staffing requirements. In 2018, the OECD reported that Ireland has one of the lowest levels of doctors per capita in Europe, at 2.9 per 1000 citizens [22] - 24% of these doctors are GPs. Comparatively, Greece has the highest at 6.6 per 1000 people, and the European average is 3.6 per 1000 people. This suggests that Ireland requires more doctors, especially when factoring in international and national healthcare targets [2, 5, 6, 12, 13, 18]. To meet current demands and achieve goals outlined by the Irish national healthcare policy “Sláintecare” [12] and the UN Sustainable Development goals to ensure affordable, universally accessible healthcare for everyone [23], more clinical staff is a requirement for the evolving health system.

In 2019, the ICGP provided evidence that GPs cite working conditions with high numbers of clinical consultations and administrative duties as reasons for moving abroad [24]. Both that report and the investigation by the ESRI [5] found that there could be a significant shortfall of GPs in Ireland in the near future. Compared to the UK and other European countries, more Irish GPs experience emotional exhaustion, a contributing factor to burnout [25]. One solution proposed is increased training of PNs, which would enable them to conduct more complex consultations, thereby increasing capacity in general practice. This supports the Department of Health recommendation to increase the number of FTE PNs by 40–89% [13] depending on system reforms.

General practice and how the public uses health services have been forced to change dramatically because of the COVID-19 emergency. There has been an increased need for remote consultations to reduce the spread of the highly contagious viral infection, which could provide a lasting solution to increase capacity in general practice [26]. Further to that, streamlined use of technology could reduce time spent on administrative duties. This unique situation has presented us with an opportunity to look at how practices might deliver care differently. However, the feasibility, acceptability and impact of all these possible solutions need to be assessed.

The ICGP is currently repeating this survey in order to quantify the changes in consultation rates and delivery method during the pandemic and once the pandemic passes, plan to replicate it at different time-points during the year. Moving forward, better mechanisms for national data collection on general practice activity and workload are critical to future-proofing primary care in Ireland especially as new technology emerges.

### Strengths and limitations

This survey had some limitations, as the sample was self-selecting and we did not capture information about patients. This means we were unable to comment on consultation rates by demographics and can only provide estimates of total consultations. Furthermore, appointments conducted in the out of hours setting were not captured, and hence our results underestimate the total workload of GPs. Data was received for each day of the normal working week, although there was a higher volume received for Monday. Another limitation is that the survey was conducted during the winter which has been shown to increase rates of respiratory illnesses and healthcare usage [27]. These factors may have resulted in an overestimation of total annual consultations. However, we did collect information from more than 500 practices spread across the country and in varying locations and estimate that this data represents 32% of all practices.

## Conclusions

We found that GPs are completing an average of 29 consultations a day or an estimated 21.4 million consultations by GPs per year in Ireland. When PN consultations are included, this increases to 29.1 million consultations taking place in Irish general practice every year. With plans to move more services into primary care [12, 13], workload is expected to increase. Through the introduction of central and standard information collection about primary care usage, it will be possible to accurately plan. Technology such as telemedicine consultations and better electronic health records could enable improved workflows and maximise access to care in Irish communities [12]. However, we need to assess how new applications of the technology [28] could bring forward change in a manner that continues to provide the Irish public with accessible, high-quality general practice care. While the need for an increased workforce is recognised [2, 5], other innovative interventions to increase the capacity of general practice are also needed to ensure high-quality care continues to be accessible in Ireland.

## Supplementary Information

**Additional file 1.** Questionnaire. The questionnaire developed to collect data for this survey.

## Data Availability

The data is available on reasonable request to the corresponding author.

## Abbreviations

ESRI: Economic and Social Research Institute
FTE: Full time equivalent
GP: General Practitioner
HSE: Health Service Executive
ICGP: Irish College of General Practitioners
OECD: Organisation for Economic Co-operation and Development
PN: Practice Nurse
WHO: World Health Organisation
