# Supplementary material for: How many general practice consultations occur in Ireland annually? Cross-sectional data from a survey of general practices
Source: BMC Fam Pract. 2021 Feb 20;22:40. doi: 10.1186/s12875-021-01377-0 (PMC7896162; doi:10.1186/s12875-021-01377-0)
Supplement: Supplementary file 1 — Additional file 1. Questionnaire. The questionnaire developed to collect data for this survey. [file 12875_2021_1377_MOESM1_ESM.pdf]

Practice Activity January 2020

Practice Activity

There has been a lot of media discussion regarding general practice and the ICGP would be grateful for your assistance in providing strong evidence so that we can inform this discussion and advocate on your behalf.

We fully appreciate that you are all very busy and we have kept the information requested to a minimum. We are asking for only one reply per practice - and this could be completed by any staff member. We have not made any questions mandatory. Of course, we hope you will complete as many as possible with the key questions relating to the number of consultations in the practice and the number of GPs and PNs on duty on the chosen day in the practice.

You can choose any typical working day - this could be yesterday or tomorrow - on which to provide data.

This survey should take about 5 minutes to complete. Thank you for taking time out of your already busy schedule to contribute data to inform this discussion and ensure accurate information is available directly from general practice.

Dr. Tony Cox, ICGP Medical Director

* 1. I confirm that I have read the introductory information and email invitation for this survey.
I consent to my data being used as part of this survey and any publications as a result of the findings.
I understand that my data will be retained on a secure server and for a short period will be held on a server located outside the E.U.
Data will only be held until the research is completed and it will then be deleted.

- ◯ Yes I consent to the above
- ◯ No I do not consent to the above, click DONE at end of survey to exit.

2. Please complete this survey in respect of any day in the surgery this week. Which day are you including data for:

- ○ Monday
- ○ Tuesday
- ○ Wednesday
- ○ Thursday
- ○ Friday

3. How many staff (GPs and Practice Nurses) were on duty on the day you are completing this survey?Please note we are asking for full time equivalents here so if someone worked half a day that is 0.5 FTE. Please enter number in deciamal format.

FTE GPs ______

FTE Practice Nurses ______

4. How many FTEs in the practice overall? Please note we are asking for full time equivalents here so if a PN usually works three days per week that is 0.6 FTE. Please enter in decimal format.

FTE GPs ______

FTE Practice Nurses ______

5. How many face to face consultations were seen in the surgery in total on the day in question: please enter in whole number format.

By GPs ______

By PNs ______

6. Do you usually leave some appointments free to accommodate urgent/same day appointments?

○ Yes ○ No

7. How many telemedicine consultations were seen on the day in question: please enter in whole number format.

By GPs ______

By PNs ______

8. How many visits by GPs were made on the day in question? Please enter in whole number format.

To homes ______

To nursing homes ______

9. On the day in question: how many hours in total did you personally work in the practice (clinical and non-clinical hours)? If you are not a GP and are completing it on behalf of the practice, please leave this question blank. Please enter in decimal format e.g. 0.5 or 2.0

10. What is the average waiting time to get a NON-URGENT appointment in your practice? Please enter in whole number format e.g. 0 or 4 etc.

For adults ________ days

For children _______ days

11. Is your practice closed to taking new patients?

	Yes	No
GMS		
Private		

12. Is your practice located in

- A City
- A Town
- A Village

13. Please indicate where your practice is located:

14. What is **the most significant** stressor for GPs at the moment? Please tick only one answer.

- Income
- Locums
- Workload
- Work/Life balance
- Regulations
- Litigation risk
- Other (please specify)
